# “Functionally” Univentricular Hearts: Impact of Pre-Natal Diagnosis

**DOI:** 10.3389/fped.2015.00015

**Published:** 2015-02-27

**Authors:** Antonio Francesco Corno

**Affiliations:** ^1^School of Medical Sciences, Universiti Sains Malaysia, Kubang Kerian, Malaysia

**Keywords:** hypoplastic left heart syndrome, peri-natal management, pre-natal diagnosis, surgical outcomes, univentricular heart

## Abstract

Within the last few decades the pre-natal echocardiographic diagnosis of congenital heart defects has made substantial progresses, particularly for the identification of complex malformation. “Functionally” univentricular hearts categorize a huge variety of heart malformations. Since no one of the patients with these congenital heart defects can ever undergo a bi-ventricular type of repair, early recognition and decision-making from the neonatal period are required in order to allow for appropriate multiple-step diagnostic and treatment procedures, either of interventional cardiology and/or surgery, on the pathway of “univentricular” heart. In the literature strong disagreements exist about the potential impact of the pre-natal diagnosis on the early and late outcomes of complex congenital heart defects. This review of the recent reports has been undertaken to better understand the impact of pre-natal diagnosis in “functionally” univentricular hearts taking into consideration the following topics: pre-natal screening, outcomes and survival, general morbidity, neurologic and developmental consequences, pregnancy management and delivery planning, resources utilization and costs/benefits issues, ethical implications, parents counseling, and interruption of pregnancy versus treatment.

## Introduction

Within the last few decades the pre-natal echocardiographic diagnosis of congenital heart defects has made substantial progresses, allowing the recognition of virtually almost all heart malformations between the 16th and 18th week of pregnancy, with a sensibility over 96% and a specificity close to 100% (1, 2).

The potential impact of pre-natal diagnosis of congenital heart defects includes:

- knowledge of the natural history of the congenital heart defects “*in utero*,”
- potential pre-natal medical or interventional cardiology therapeutic interventions in the case of diagnosed heart failure, arrhythmias, or malformations with poor neonatal prognosis,
- safer management of the pregnancy itself,
- organization of the peri-natal period in or close to institutions with facilities available for the immediate management in the case of life-threatening heart malformations,
- parental counseling.

“Functionally” univentricular hearts categorize a huge variety of heart malformations, including but not limited to mitral atresia, tricuspid atresia, hypoplastic right or left heart, pulmonary atresia with intact ventricular septum and hypoplastic right ventricle, aortic atresia, double outlet right ventricle with hypoplastic right or left ventricle, complete atrio-ventricular septal defect with unbalanced ventricles, double inlet right or left ventricle.

Since no one of the patients with these congenital heart defects can ever undergo a bi-ventricular type of repair, early recognition and decision-making from the neonatal period are required in order to allow for appropriate multiple-step diagnostic and treatment procedures, either of interventional cardiology and/or surgery, on the pathway of “univentricular” heart.

In the literature strong disagreements exist about the potential impact of the pre-natal diagnosis on the early and late outcomes of complex congenital heart defects. This review of the recent reports has been undertaken to better understand the impact of pre-natal diagnosis in “functionally” univentricular hearts.

Of course it has to be taken into account the difficulty of having meaningful inferences from the literature, because of different inclusion criteria, relative small numbers of patients, different peri-operative managements, different end-points, and frequently insufficient statistical analysis.

## Literature Review

The reported studies have been analyzed, taking into consideration the impact of the pre-natal diagnosis on the following topics:

- pre-natal screening,
- outcomes and survival,
- general morbidity,
- neurologic and developmental consequences,
- pregnancy management and delivery planning,
- resources utilization and costs/benefits issues,
- ethical implications, parents counseling, interruption of pregnancy versus treatment.

Since any of the above topics presents direct implications with most of the other, the analysis has been performed trying to separately concentrate on each of the specific matters.

## Pre-Natal Screening

The pre-natal echographic screening is, by necessity, the first step in the process of pre-natal diagnosis and management of complex congenital heart defects, and therefore several reports are available in the literature, focused on the pre-natal screening, with considerations on general and specific issues (3–24).

### General non-cardiac issues

A higher incidence of chromosomal disorders has always been found in fetuses with complex congenital heart defects (4, 8, 16), as well as reduced fetal body weight and growth (6, 22), associated non-cardiac malformations (16), and the presence of situs inversus or heterotaxy (18).

### General cardiac issues

The incidence of complex congenital heart defects, including “functionally” univentricular hearts, has been observed as higher in the fetal pre-natal cardiac screening than in the post-natal diagnosis (4, 6, 7, 10, 12, 18, 21, 23).

The pre-natal cardiac screening can also been useful to detect fetal arrhythmias (12). Of course, the pre-natal diagnosis of complex congenital heart defect has opened the possibility of investigating potential fetal interventions (11).

### Specific cardiac issues

The most important information provided by the pre-natal echocardiographic screening is relative to the morphology, size, shape, and function of the ventricular chambers, even given the known difficulties in evaluating the ventricular performance based only on pre-natal echocardiography (3, 13, 14, 19, 20).

Data on the morphology and functioning of the right atrio-ventricular valve are also relevant (3, 18, 20).

In the specific cases of pre-natal diagnosis of hypoplastic left heart, the presence of restrictive inter-atrial communication, and the type and direction of the flow through the inter-atrial communication itself (3, 5, 8, 9, 15), as well as the responsiveness of the fetal pulmonary vasculature to maternal hyperoxygenation (5), are indispensable for a prognostic evaluation and for the organization of the immediate post-natal treatment. Also the pre-natal evidence of endocardial fibroelastosis has clear negative prognostic implications (20, 24).

Useful information on the expected pathophysiology are the type and direction of flow through the patent ductus arteriosus (17), and particularly the presence of obstructed or retrograde flow through the aortic arch (3, 19).

A recent report proved the feasibility of pre-natal echocardiographic demonstration of the presence of ventriculo-coronary connections (24).

## Survival

Survival is of course the most important parameter when evaluating the impact of the pre-natal diagnosis on the outcomes, and this issue has been addressed by many studies (3, 6, 9, 23, 25–32).

While for a relatively simple congenital heart defect, such as aortic coarctation, the pre-natal diagnosis can contribute to improve the outcomes, including the survival (28), for a more complex defect, such as transposition of the great arteries, disagreement exists on the impact of the pre-natal diagnosis, going from reporting improved pre-operative as well as post-operative survival (25), to reporting only improved pre-operative condition but without any improvement of the post-operative outcomes (26).

When dealing with the most complex congenital heart defects, such as “functionally” univentricular hearts, because of the presence of multi-factorial elements influencing the outcomes, the discrepancy on the opinions about the impact of pre-natal diagnosis on survival becomes even more evident (3, 6, 9, 23, 27, 29–32).

In the past clinical studies failed to show an improved overall survival when the diagnosis of hypoplastic left heart syndrome was made pre-natal (33, 34).

In the more recent period, with substantially improved the peri-operative care, a dichotomy clearly divided the reports demonstrating improved survival with pre-natal diagnosis (3, 9, 30–32) from the studies showing similar survival between cases with pre-natal and post-natal diagnosis (6, 23, 26, 27, 29).

Despite this discrepancy regarding the survival, almost all studies showed the improved pre-operative conditions obtained thanks to the pre-natal diagnosis and the subsequent timely transfer to tertiary referral hospital for intensive care treatment. General agreement exists on the observations that prompt referral after pre-natal diagnosis allows reducing the pre-operative metabolic acidosis and the subsequent need for bicarbonate administration, minimizing the needs for pre-operative inotropic support, reducing the incidence and degree of ventricular dysfunction and right atrio-ventricular valve regurgitation, and almost abolishing the occurrence of end-organs dysfunction (3, 6, 26, 27, 29–32). The pre-natal diagnosis allows also early intervention in the case of identification of restrictive inter-atrial communication (9).

It is very interesting to note that, despite in the vast majority of the reported studies, the pre-operative conditions were significantly better in the cases with pre-natal diagnosis versus the post-natal diagnosis, this improvement failed to provide improved operative survival in about half of the clinical observations.

The impact of the pre-natal diagnosis has not yet been evaluated on the outcomes of the hybrid approach for hypoplastic left heart syndrome, with bilateral pulmonary artery banding and stenting of the patent ductus arteriosus. This treatment option is increasingly considered or used in the recent years in the patients considered with high-risk for the conventional surgical management (35–43).

## General Morbidity

The comparison between the two groups with pre-natal and post-natal diagnosis with regard to the post-operative morbidity showed discrepancies exactly as for the survival (3, 6, 26, 29, 31).

Few studies showed reduced post-operative morbidity in the group with pre-natal diagnosis (3, 6, 31). The reasons to support the decreased morbidity were the better pre-operative conditions, particularly because of the reduced metabolic acidosis (3, 31), reduced duration of mechanical ventilation (6), and reduced need for emergency operation (6), and the observations of better post-operative ventricular function (3, 31).

Other researchers failed to observe reduced morbidity in the group of patients with pre-natal diagnosis (26, 29).

## Neurologic and Developmental Consequences

Among the studies investigating the early and late neurocognitive development in children with complex congenital heart defects, very few had taken into consideration the impact of pre-natal diagnosis (27, 30, 44, 45).

In fetuses with hypoplastic left heart syndrome, a significant decrease in head growth and chronic diffuse white matter injury of variable severity have been reported (44). The pre-natal diagnosis has been associated with reduced incidence of adverse peri-operative neurologic events (27), as well as reduce incidence, and lesser severity of neurocognitive deficits (45).

Despite it is well known that children with complex congenital heart defects present an increased prevalence of central nervous system abnormalities, due to inadequate development, pre-natal ischemia, post-natal cyanosis, and exposure to cardio-pulmonary bypass, it seems that pre-natal diagnosis, allowing the reduction of the peri-operative multi-organ injuries, can also improve the long-term beuroligic outcome (27, 30, 45).

## Pregnancy Management and Delivery Planning

Quite evident is the agreement on the positive impact of pre-natal diagnosis of complex congenital heart defect, particularly “functionally” univentricular heart, in the management of the pregnancy and the appropriate planning of the delivery (3, 7, 17, 46–49).

After pre-natal echocardiographic diagnosis of “functionally” univentricular heart, a maternal-fetal care program can be established, with the pregnancy follow-up and the mode of elective delivery, either cesarean or vaginal with induced or spontaneous labor, timely planned and organized in a tertiary referral hospital. This allows appropriate multi-disciplinary management of the neonate from the delivery room to the operating room, including echocardiographic confirmation of the cardiac diagnosis shortly after birth, and immediate transfer to the neonatal intensive care unit for prompt administration of prostaglandins, maintenance of normal acid-base status, and tracheal intubation with mechanical ventilation to provide and maintain adequate balance between the systemic and the pulmonary circulation (3, 17, 46–49).

## Resources Utilization and Costs/Benefits Issues

In an era of money-driven medicine, the efforts toward cost containing have reached also the potential impact of pre-natal diagnosis of complex congenital heart defects, including “functionally” univentricular hearts (50, 51).

The costs of training ultrasound technicians and make them capable to perform pre-natal diagnosis of complex congenital heart defects have been compared with the costs of organizing the emergency transports of neonates with post-natal diagnosis to specialized care centers. The results showed a substantial saving, considering the costs of training the ultrasound technicians plus the organization of elective transfer for delivery to a tertiary referral center in the case of pre-natal diagnosis of complex congenital heart defect versus emergency transportation and care after post-natal diagnosis of similar heart malformations (50). The saving was directly correlated with the detection rate, with considerable increase for each day of exposure of the ultrasound technicians to the specific training (50).

## Ethical Implications, Parents Counseling, Interruption of Pregnancy Versus Treatment

All these points, strictly correlated, are not quantifiable in scientific or mathematic terms, such as the sensitivity of the pre-natal screening or the percentage of survival. A very large number of studies have tried to cover the matter, taking in consideration all the various aspects, and the unavoidable inter-relationships (3, 8, 16, 52–74).

First of all, in this review all cases with the pre-natal diagnosis of “functionally” univentricular heart have been taken into consideration. In this regard, despite decent medium and long-term results obtained from few decades with the univentricular type of repair (75–79), the concept of “intractable” congenital heart defect is still associated with the diagnosis of univentricular heart, because of the hazardous long-term results (57, 69). “Intractable” (57) or “uncorrectable” (69) has been defined the heart malformations not suitable to bi-ventricular repair, and therefore where the only option available for treatment remains multi-staged complex palliation programs toward univentricular type of repair or heart transplant. The only alternative possible in these cases is abstention from any treatment or compassionate care (57, 69).

With regard to the option of heart transplant, evident discrepancies exist between the reported data in relationship with the timing of the heart transplant, if the option was utilized as first stage, after failed first stage Norwood type of procedure, after the second stage (bidirectional Glenn anastomosis), or after failed Fontan circulation (80–83).

In fact it has been reported (3, 8, 16, 53, 59, 60, 64, 66, 67, 69, 70, 73) that following the pre-natal diagnosis of complex congenital heart defect not suitable for bi-ventricular repair, the decision to terminate the pregnancy or for compassionate care has been taken with percentages varying from 8 (64) through 16 (8, 16), 30 (59, 60), 33 (3), up to 47% (73).

The decision for abstention from treatment, very difficult for all couples of parents, was generally justified by one or more of the following reasons: to avoid a described poor hospital survival and diminished quality of life, to prevent the child from suffering (53, 59), to reduce the stress to the parents themselves and to the siblings (53, 69), and because of the presence of chromosomes disorders or major extra-cardiac malformations (73).

In almost all cases the decision of the parents has been influenced by availability of pre-natal diagnosis of complex congenital heart defect (16, 53, 59, 70, 73). Because of the striking importance of the impact of pre-natal diagnosis on the decision-making process, general agreement exists that following pre-natal diagnosis of complex congenital heart defect the parents should be offered a comprehensive multi-disciplinary pre-natal evaluation, including karyotyping, detailed intra-cardiac and extra-cardiac assessment, in order to obtain the most possible accurate prognosis for early and long term outcomes (8, 16, 52, 59–62, 64, 66, 69, 74). It is vital to give the parents and their families all information and support to prepare their heart and mind (69).

It is easy to agree about the importance of a multi-disciplinary team to assist the families after pre-natal diagnosis of complex congenital heart defect. Much more difficult is to define the “who, what, when, where and how” relative to the communication of the information to the parents.

The information of pre-natal diagnosis of complex congenital heart defect is unavoidably followed by a major psychological crisis of the parents and their family: the state of shock, the short time available to make decisions, the frequently contradictory information available about early and long-term outcomes, limit to the parents the possibility for autonomous choices (53). Furthermore, autonomous decisions may be neither possible nor desirable for all the parents (53).

The parents’ counseling, already difficult because of the problem of communications between caregivers and families, can be further complicated by the influence of religion, culture, individual cognitive, emotional, and social processes (54, 55, 69, 71). As a matter of fact, cross-cultural patient care is an issue challenging the healthcare providers: caring for patients who reject some biomedical treatments because of religious or cultural reasons requires knowledge of that person’s beliefs to establish an effective treatment (55, 71).

Another well-known difficulty of the parental counseling is due to the fact that nowadays the parents have easy access, through internet, to the best results reported by the best hospitals, while the same results are most probably not obtained in the local hospital. At this point it is very uneasy for a doctor to confess that the results of the local hospital are less satisfactory that the gold standard reported on the net.

During the process of parents’ counseling, when considering the information received or perceived by the parents on the potential or expected outcomes, well known is the influence or the type and quality of messages received from the caregivers. While it is quite intuitive that the influence by optimistic physicians at surgical departments seemed important for a choice orientated toward surgery, less expected is the observation that the opinions offered by the professionals have offended some parents (61, 62, 67–69, 74).

The importance of doctors’ belief, varying from the two extremes, optimism regarding the potential long-term outcomes, or serious suggestion to consider the option of termination of pregnancy, has determinant implications on the parental perceptions and their subsequent decision-making process (56, 65, 67, 70, 72).

Also the opinion of the patients, and their families, should be taken into consideration, because there are certainly different degree of acceptance of a limited quality of life by different individuals and families.

Doctor’s recommendations for treatment are consistent with selecting the option that maximizes the chance of the best outcome, without necessarily minimizing the chance of the worst outcome; even their beliefs about likely outcomes vary according to the patient group they encountered (56).

The risk communication is particularly problematic when the evidence base is limited or when there are several options with many possible outcomes (56). Finally, because of the complexity of the pre-natal diagnosis and the subsequent huge variety among the potential long-term outcomes, very difficult is for the caregivers is to maintain a professional attitude, not influenced by the personal feelings and opinions.

Not easy is to find an answer to the question: “What if it were your child?” (58). An attempt to answer the above question has been made with an “Audience Response Survey” at the first joint meeting of the “Congenital Heart Surgeons Society” (of North America) and the European Association Congenital Heart Surgeons, collecting the answer form 73 experienced congenital heart surgeons (63). The results of the survey are represented in Table 1. Evident is the striking difference between the suggestion given by the surveyed heart surgeons if the pre-natal diagnosis was done for a patient rather than for a family member.

**Table 1:** **Results of the “Audience Response Survey”**.

Options	Patient (%)	Family member (%)
Norwood	73	26
Heart transplant	0	1
Compassionate care	6	5
Pregnancy interruption	21	68

## Conclusion

The impact of pre-natal diagnosis of complex congenital heart defects where the only treatment option is a univentricular heart type of repair has dramatic implications for the parents, their families, and all the involved caregivers.

The huge incidence of variable morphologic characteristics and subsequent pathophysiologic patterns, the potential association of chromosomal disorders, associated intra-cardiac and extra-cardiac malformation, and the several clinical reports with evolving short- and long-term outcomes make the parents counseling and the subsequent decision-making process extremely difficult.

Better information, and therefore suggestions, could come from prospective data collection of the outcomes obtained with the currently available peri-operative techniques.

## Conflict of Interest Statement

The author declares that the research was conducted in the absence of any commercial or financial relationships that could be construed as a potential conflict of interest.
